# Supplementary material for: Considerations in evaluating equipment-free blood culture bottles: A short protocol for use in low-resource settings
Source: PLoS One. 2022 Apr 25;17(4):e0267491. doi: 10.1371/journal.pone.0267491 (PMC9037908; doi:10.1371/journal.pone.0267491)
Supplement: S1 File — (DOCX) [file pone.0267491.s001.docx]

# **S1 File: Report on head-to-head comparison of horse blood with human blood for BCB validation studies**

**Rationale**

To perform a laboratory validation or verification of blood culture bottles, blood culture bottles are usually inoculated with blood and bacteria. To simulate real-life conditions as closely as possible, human blood is preferable for these experiments. However, the use of human blood for blood culture bottle experiments is hindered by the limited availability of left-over blood in blood banks and ethical issues regarding use of blood from volunteers. Moreover, even if a steady supply from the blood bank can be achieved, the blood used for validation will often come from different volunteers, compromising standardization of the experiments, as differences in performance may be attributable to differences in the blood (*e.g.* different levels of plasma components such as antibodies), rather than differences in the bottles or bacterial strains. The use of animal blood, sampled in standardized conditions, is therefore an attractive option to organize and standardize large blood culture bottle evaluations.

We performed an experiment comparing yield and bacterial speed of growth (time-to-positivity) in horse blood to yield and speed of growth in human blood, with an automated incubator (BacT/ALERT, bioMérieux, Marcy-l’Etoile, France).

To evaluate the differences in visual signs of growth between horse blood and human blood, a small number of biphasic manual blood culture bottles were inoculated with both human and horse spiked blood. Signs of growth were compared.

**Methods**

The bottles used for this validation were BacT/ALERT FA Plus Aerobic bottles (ref 410851, bioMérieux, Marcy-l’Etoile, France), the BacT/ALERT PF Plus Pediatric bottle (ref 410853, bioMérieux) and Autobio Bi-State blood culture bottle (ref M0601; Autobio Diagnostics Co, Zhengzhou, China). A BacT/ALERT automated incubator (BacT/ALERT® 3D 120) was used for the BacT/ALERT bottles.

Spiking of the blood was done with ATCC strains and strains obtained from external quality assessments (EQA) sent out by the Belgian Institute of Public Health (Sciensano). The strains tested were the following:

- - *Escherichia coli* ATCC 25922
  - *Staphylococcus aureus* ATCC 25923
  - *Salmonella* Typhimurium M/10452
  - *Haemophilus influenzae* ATCC 49247
  - *Streptococcus pneumoniae* ATCC 49619
  - *Streptococcus agalactiae* ATCC 27956
  - *Klebsiella pneumoniae* ATCC 700603
  - *Burkholderia cepacia* M2514
  - *Acinetobacter baumannii* 2000/1
  - *Pseudomonas aeruginosa* ATCC 27853

A dilution series of each strain was done, starting from a 0.5 McFarland dilution (40 x 10^4^ dilution). For adult bottle formulations, an estimated end concentration of 5 CFU/ml of blood was prepared. Three BacT/ALERT FA Plus bottles were inoculated with 10 ml of the spiked human blood and three bottles were inoculated with 10 ml of the spiked horse blood. For pediatric bottle formulations, an end concentration of 10 CFU/ml of blood was prepared. Of the BacT/ALERT PF Plus bottles, three bottles were inoculated with 2 ml of the spiked human blood and three bottles were inoculated with 2 ml of the spiked horse blood. Of the Autobio bottles, one bottle was inoculated with 2 ml of the spiked human blood and one with 2 ml of the spiked horse blood (both in pediatric concentrations). Manual BCB were incubated in a standard incubator at 35°C and inspected twice daily for signs of growth for the first two days of incubation. The BacT/ALERT bottles were placed in the BacT/ALERT incubator. Agar of the biphasic BCB was flooded at the moment of inoculation and again after 48 hours of incubation if no significant growth of colonies was present at that time. Time-to-detection, as stored by the automate, was noted for the automated bottle. Time and date of visual signs of growth in the broth and on the agar were noted, and the type of growth was noted. Subculture on blood agar of all bottles was done at signs of positivity in broth/agar/automate. Two-sided paired student T-test was used to analyze the difference in mean time-to-detection between horse blood and human blood. Wilcoxon signed-rank test (for paired samples) was used to analyze the difference in median time-of-detection between horse blood and human blood. Data analysis was done with Excel Analyse-It software.

**Results**

In total, 120 BacT/ALERT bottles and 20 Autobio bottles were inoculated with spiked blood and incubated. Half of these were inoculated with spiked horse blood, half with spiked human blood. For the BacT/ALERT bottles, half of the bottles were pediatric formulations (PF Plus bottles), half were adult formulations (FA Plus bottles). All BacT/ALERT bottles showed growth within 24 hours of incubation. All Autobio bottles had shown signs of growth after 2 nights of incubation. Yield was therefore 100% for all inoculated bottles, regardless of blood type.

**S1 Table 1: Results for mean, median, standard deviation and interquartile range of time-to-detection in BacT/ALERT bottles; all species combined**

|  | Horse blood (n = 60) | | | Human blood (n = 60) | | | p-value difference horse-human blood | | |
| --- | --- | --- | --- | --- | --- | --- | --- | --- | --- |
|  | Pediatric | Adult | **Overall** | Pediatric | Adult | **Overall** | Pediatric | Adult | **Overall** |
| Mean time-to-detection | 14h02 | 13h54 | **13h58** | 14h49 | 14h17 | **14h33** | 0,07 | 0,48 | **0,09** |
| Standard deviation | 2h22 | 2h29 | 2h25 | 3h26 | 3h35 | 3h29 |  |  |  |
| Median time-to-detection | 13h09 | 13h34 | **13h20** | 13h20 | 12h34 | **12h59** | 0,22 | 0,42 | **0,82** |
| Interquartile range | 12h10 – 14h45 | 11h50 – 15h17 | 12h05 – 14h56 | 12h11 – 16h40 | 11h48 – 16h19 | 12h09 – 16h26 |  |  |  |

Mean time-to-detection was shorter for horse blood than for human blood; the opposite was true for the median time-to-detection. Standard deviation and interquartile range show that data were more dispersed for human blood than horse blood, suggesting a more skewed distribution of the time-to-detection for human blood than horse blood. Differences between horse blood and human blood in central moments (mean and median) range from 11 minutes (median time-to-detection pediatrics) to 60 minutes (median time-to-detection adults). These differences are not statistically significant; it must be mentioned that this study was not powered to show small differences, the minimum difference that could be demonstrated with 80% power was 2h30. A difference of this magnitude would not be clinically significant for most clinical laboratories. Maximum time-to-positivity was 19:22 for horse blood and 22:32 for human blood, showing that all bottles were positive within 24 hours.

All inoculated Autobio bottles showed first sign of growth in broth after 1 night of incubation (n = 20), both for human blood and horse blood.

**S1 Table 2: results of growth in manual, biphasic blood culture bottles (Autobio). These bottles consist of a broth (liquid phase) and an agar slant (solid phase).**

|  | Total number tested | First sign of growth in broth | | First sign of growth on agar slant* | |
| --- | --- | --- | --- | --- | --- |
|  |  | Day 1 morning | Day 1 afternoon | Day 1 | Day 2 |
| Human blood | 10 | 10 | 0 | 4 | 5 |
| Horse blood | 10 | 9 | 1 | 4 | 5 |

* one species (*Acinetobacter baumannii*) did not show growth on agar slant

One bacterial species (*Pseudomonas aeruginosa*) showed a film on the agar one day earlier for human blood than for horse blood. Another (*Haemophilus influenzae*) showed visible growth on agar one day earlier for horse blood than for human blood. For *Burkholderia cepacia*, turbidity in the broth was noted in the afternoon in horse blood, and already in the morning for horse blood. For *Klebsiella pneumoniae*, horse blood showed hemolysis but human blood did not.

These were the only differences noted between horse blood and human blood. It must be noted here that the sample size was small (10 bottles in each group).

**Conclusion**

Based on these findings, any differences in bacterial growth between horse blood and human blood are small and clinically non-significant. Therefore, use of horse blood for blood culture experiments is acceptable.
