# Supplementary material for: Considerations in evaluating equipment-free blood culture bottles: A short protocol for use in low-resource settings
Source: PLoS One. 2022 Apr 25;17(4):e0267491. doi: 10.1371/journal.pone.0267491 (PMC9037908; doi:10.1371/journal.pone.0267491)
Supplement: S2 File — (DOCX) [file pone.0267491.s002.docx]

# **S2 File: Examples of worksheets to be used in BCB validation study**

Work sheet 1 **Blood culture bottle reference validation: worksheet per strain**

Date and time of incubation: _________________________

Strain inoculated: __________________________________

Lot number bottle under evaluation: A (adult): _______________________________; P (pediatric): _______________________________

Lot number reference bottle: A (adult):____________________________; P (pediatric): _______________________________

colony count plate 1: colony count plate 2: colony count plate 3: mean colony count:

| **Bottle nr.** | **Bottle type and nr** | **Date and time of first visual signs of positivity** | **For automated: time to positivity** | **Result Gram stain** | **Growth on blind subculture on day 1**  **(Y/N)**  **(only for manual bottles)** | **Growth on subculture at moment of positivity**  **(Y/N)**  **(Only when blind subculture negative)** | **Growth on terminal subculture**  **(Y/N)**  **(Only when other subcultures negative)** | **Remarks** |
| --- | --- | --- | --- | --- | --- | --- | --- | --- |
| 1. | Reference bottle 1 A |  |  |  |  |  |  |  |
| 2. | Reference bottle 2 A |  |  |  |  |  |  |  |
| 3. | Reference bottle 3 A |  |  |  |  |  |  |  |
| 4. | Evaluated bottle 1 A |  |  |  |  |  |  |  |
| 5. | Evaluated bottle 2 A |  |  |  |  |  |  |  |
| 6. | Evaluated bottle 3 A |  |  |  |  |  |  |  |
| **Bottle nr.** | **Bottle type and nr** | **Date and time of first visual signs of positivity** | **For automated: time to positivity** | **Result Gram stain** | **Growth on blind subculture on day 1**  **(Y/N)**  **(only for manual bottles)** | **Growth on subculture at moment of positivity**  **(Y/N)**  **(Only when blind subculture negative)** | **Growth on terminal subculture**  **(Y/N)**  **(Only when other subcultures negative)** | **Remarks** |
| 7. | Reference bottle 1 P |  |  |  |  |  |  |  |
| 8. | Reference bottle 2 P |  |  |  |  |  |  |  |
| 9. | Reference bottle 3 P |  |  |  |  |  |  |  |
| 10. | Evaluated bottle 1 P |  |  |  |  |  |  |  |
| 11. | Evaluated bottle 2 P |  |  |  |  |  |  |  |
| 12. | Evaluated bottle 3 P |  |  |  |  |  |  |  |

Work sheet 2

Strain inoculated: __________________________________

**Type of growth in broth**

| **Bottle nr.** | **Bottle code** | **Date and time of first sign of growth in broth** | **Date of turbidity** | **Date of gas production** | **Date of puff balls** | **Date of pellicle/film on broth** | **Date of hemolysis** | **Date of colour change indicator (if applicable)** |
| --- | --- | --- | --- | --- | --- | --- | --- | --- |
| 4. | Evaluated bottle 1 A |  |  |  |  |  |  |  |
| 5. | Evaluated bottle 2 A |  |  |  |  |  |  |  |
| 6. | Evaluated bottle 3 A |  |  |  |  |  |  |  |
| 10. | Evaluated bottle 1 P |  |  |  |  |  |  |  |
| 11. | Evaluated bottle 2 P |  |  |  |  |  |  |  |
| 12. | Evaluated bottle 3 P |  |  |  |  |  |  |  |

**Types of growth on agar (if applicable)**

| Bottle nr. | Bottle code | Date and time of first sign of growth on agar | Date of gas bells in agar | Date of growth barely visible | Date of film on agar | Date of confluent growth | Date and time of single colonies | Date of pickable colonies/growth (sufficient growth) |
| --- | --- | --- | --- | --- | --- | --- | --- | --- |
| 4. | Evaluated bottle 1 A |  |  |  |  |  |  |  |
| 5. | Evaluated bottle 2 A |  |  |  |  |  |  |  |
| 6. | Evaluated bottle 3 A |  |  |  |  |  |  |  |
| 10. | Evaluated bottle 1 P |  |  |  |  |  |  |  |
| 11. | Evaluated bottle 2 P |  |  |  |  |  |  |  |
| 12. | Evaluated bottle 3 P |  |  |  |  |  |  |  |
