# Supplementary material for: Considerations in evaluating equipment-free blood culture bottles: A short protocol for use in low-resource settings
Source: PLoS One. 2022 Apr 25;17(4):e0267491. doi: 10.1371/journal.pone.0267491 (PMC9037908; doi:10.1371/journal.pone.0267491)
Supplement: S3 File — (DOCX) [file pone.0267491.s003.docx]

# **S3 file: Ease of Use assessment**

**Importance of ease of use assessment**

1. **Ease of use of blood sampling**

A very thick or rigorous septum complicates blood culture sampling, as considerable pressure has to be put on the needle to pierce the septum, increasing the risk of needlestick injuries and infectious spills. Moreover, a very rigorous septum may tempt the healthcare staff to open the BCB (in case of screw caps) to inject the blood, increasing the risk of environmental contamination.

The use of butterfly needles for blood culture sampling lowers the risk of needlestick injury (1). To use butterfly needles, adapters fitting the BCB “head” are needed to allow direct connection of the tube to the BCB. For the most commonly used BCB, these adapters are available (*e.g.,* BacT/ALERT and BACTEC bottles), but they may not fit other BCB. The compatibility of the BCB with commercially available adapters should be evaluated.

1. **Visual evaluation of growth in the BCB**

To better assess visual growth, BCBs should be manufactured in completely transparent plastic or glass. Scratches or large non-transparent labels may affect visual assessment. BCB designed for automated systems have an indicator that detects CO_2_ changes in the blood-broth mixture. For BacT/ALERT BCBs, this indicator is a chromogenic indicator. For BACTEC BCBs, this indicator is fluorescent. Visual evaluation of these indicators (using a fluorescent lamp in case of BACTEC bottles) has been used in some studies, mainly in LRS, with satisfactory results (2–4). Other possible aids to ease visual growth detection are pH indicators added to the broth, or the slant of biphasic BCB (BCB consisting of a broth and agar slant).

1. **Labelling of BCB**

Necessary information, such as product name, lot number and expiration date should be present on the BCB. Preferably, labelling is transparent. There should be sufficient space left on the BCB to minimally write name and patient number, date and time of sampling, site of sampling (left arm, right arm, other), initials of the person handling the sample, and a laboratory barcode if needed, all of this without compromising visual assessment of growth. A peel-off label with an identical number or barcode as the main BCB label is a valuable addition, as it can be glued to the request form to reduce errors in patient and sample identification. A mark on the BCB indicating recommended filling volume is also desirable.

1. **BCB material should be in line with biosafety requirements (follow country guidelines)**

As mentioned above, thick septa and insufficient vacuum may increase risk of needlestick injury. Plastic is preferred over glass, because it weighs less (important when BCBs have to be shipped overseas). Moreover, breakage occurs less frequently and is not associated with sharps that can lead to injuries. The World Health Organization recommends inactivation of laboratory Biological Safety Levels 2 and 3 waste by autoclaving before transport for incineration and subsequent disposal (5). Glass, polycarbonate and polypropylene withstand autoclavation (6), but polyethylene terephthalate (PET) bottles show considerable deformation on autoclavation, so protection for spillage (autoclave bag) must be foreseen.

**Survey for ease of use assessment**

**How would you rate ease of use of the blood culture bottles, in the following areas? Please circle your response.**

|  | Strongly disagree | Disagree | Neutral | Agree | Strongly agree | Not applicable |
| --- | --- | --- | --- | --- | --- | --- |
| Material are easy to see through, no scratches | 1 | 2 | 3 | 4 | 5 | NA |
| It is easy to perforate the septum with a needle | 1 | 2 | 3 | 4 | 5 | NA |
| The septum is protected from dust with an easily removable cover | 1 | 2 | 3 | 4 | 5 | NA |
| It is easy to access colonies on agar slant (*only for biphasic bottles*) | 1 | 2 | 3 | 4 | 5 | NA |
| Good grip with gloves; bottles don’t slip | 1 | 2 | 3 | 4 | 5 | NA |
| It is easy to label and write on bottle | 1 | 2 | 3 | 4 | 5 | NA |
| There is sufficient space on the bottle to label it with patient information | 1 | 2 | 3 | 4 | 5 | NA |
| It is easy to detect growth in bottle | 1 | 2 | 3 | 4 | 5 | NA |
| The vacuum is sufficient for passive filling of the bottle | 1 | 2 | 3 | 4 | 5 | NA |

**Do you have any additional comments about ease of use?**

……………………………………………………………………………………………………………………………………………………………………..

……………………………………………………………………………………………………………………………………………………………………..

……………………………………………………………………………………………………………………………………………………………………..

**The following checklist refers to general characteristics of the bottle. Tick the box that applies.**

|  | **Yes** | **No** | **Unknown** |
| --- | --- | --- | --- |
| The bottle is compatible with an adapter for butterfly needles | □ | □ | □ |
| There is a growth detection aid present in or on the bottle (agar slant, colour indicator, pH indicator, …) | □ | □ | □ |
| There is a peel-off label with unique bottle number or barcode present on the bottle | □ | □ | □ |
| Product name and reference number are present on the bottle | □ | □ | □ |
| Lot number is present on the bottle | □ | □ | □ |
| Expiration date is present on the bottle | □ | □ | □ |
| The recommended filling volume is indicated on the bottle | □ | □ | □ |
| The bottle is made of polycarbonate or polypropylene (compatible with autoclavation) | □ | □ | □ |
| The bottle is made of glass | □ | □ | □ |

**References S3**

1. Ombelet S, Barbé B, Affolabi D, Ronat J-B, Lompo P, Lunguya O, et al. Best Practices of Blood Cultures in Low- and Middle-Income Countries. Front Med. 2019;6(June).

2. Peeters M, Ombelet S, Chung P, Tsoumanis A, Lim K, Long L, et al. Slow growth of Burkholderia pseudomallei compared to other pathogens in an adapted blood culture system in Phnom Penh, Cambodia. J Med Microbiol [Internet]. 2019;1–8. Available from: https://www.microbiologyresearch.org/content/journal/jmm/10.1099/jmm.0.001011.v1

3. Andrews JR, Prajapati KG, Eypper E, Shrestha P, Shakya M, Pathak KR, et al. Evaluation of an Electricity-free, Culture-based Approach for Detecting Typhoidal Salmonella Bacteremia during Enteric Fever in a High Burden, Resource-limited Setting. PLoS Negl Trop Dis. 2013;7(6).

4. Archibald LK, Donald LCMC, Addison RM, Knight CMC, Byrne T, Dobbie H, et al. Comparison of BACTEC MYCO / F LYTIC and WAMPOLE ISOLATOR 10 ( Lysis-Centrifugation ) Systems for Detection of Bacteremia , Mycobacteremia , and Fungemia in a Developing Country. J Clin Microbiol. 2000;38(8):2994–7.

5. WHO. Laboratory biosafety manual. Third edition. World Health Organization [Internet]. 3rd ed. World Health Organization. Geneva; 2004. 1–178 p. Available from: http://books.google.com/books?hl=en&amp;lr=&amp;id=qVHfjFlNjzwC&amp;oi=fnd&amp;pg=PP9&amp;dq=Laboratory+biosafety+manual&amp;ots=Qw_FLuZChi&amp;sig=xgd8x9nOBNLlDE5wsH-WAZP6Kw0

6. InterFocus. Guide to autoclaving plastics and glass. https://www.mynewlab.com/blog/a-guide-to-autoclaving-plastics-and-glass/.
